# Role of Inorganic Fillers on the Physical Aging and Toughness Loss of PLLA/BaSO_4_ Composites

**DOI:** 10.1021/acsapm.3c02112

**Published:** 2023-11-01

**Authors:** Xabier Larrañaga, Jose R. Sarasua, Ester Zuza

**Affiliations:** Department of Mining-Metallurgy Engineering and Materials Science & POLYMAT, Faculty of Engineering, University of the Basque Country (UPV/EHU), Alameda de Urquijo s/n, Bilbao 48013, Spain

**Keywords:** poly(l-lactide), physical aging, toughness, barium sulfate, composites, molecular mechanics, debonding

## Abstract

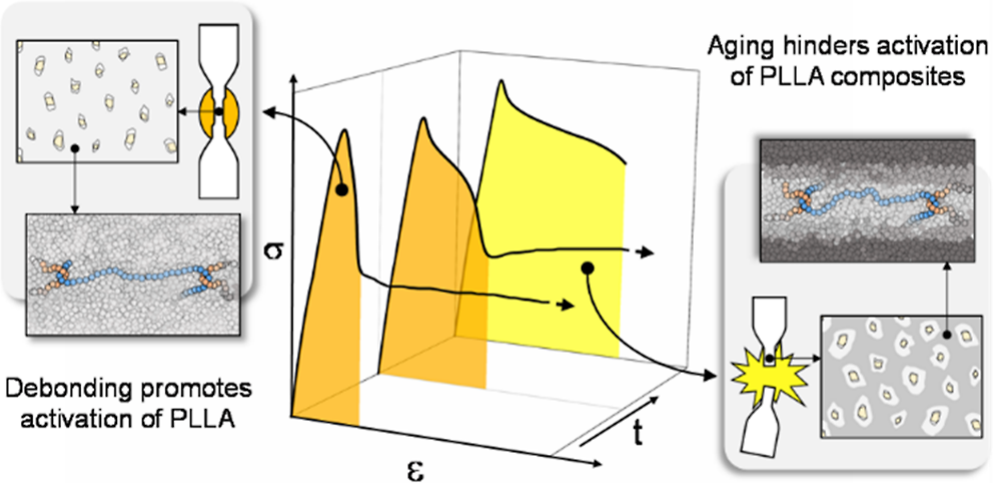

The addition of inorganic fillers has been reported to increase the toughness of poly(l-lactide) (PLLA), but the effect of physical aging in such composites has been neglected. The present work discusses the effect of the still ongoing segmental relaxation in PLLA-based composites filled with BaSO_4_ inorganic particles in regard of the filler quantity. By means of differential scanning calorimetry, X-ray diffraction, and tensile testing of progressively aged PLLA filled with particles ranging from 0.5–10 wt %, we observed an increase in the mechanical energy required to activate the plastic flow of the primary structure in the PLLA matrix, which resulted in the embrittlement of the majority of composites upon enough aging. Results further clarify the role of debonding in the activation process of PLLA, and the behavior of the composite is described at the segmental level. Only an addition of 10% of particles has effectively preserved a ductile behavior of the samples beyond 150 aging days; therefore, we strongly remark the significance of studying the effect of physical aging in such composites.

## 1 Introduction

Medical devices like prosthetics and fixation anchorages must fulfill, among other properties, a series of mechanical requirements to ensure reliable performance during implantation of the part and its service life, primarily due to the dreadful consequences that can carry a malfunctioning medical implant. The undesired rupture of the device due to external loads or impacts shall be avoided at all costs for which high-toughness materials are requested for these applications. Moreover, stiffness is also a critical property in tissue regeneration engineering, since matching the elastic modulus of the replaced tissue decreases the risk of stress shielding, which makes the newly grown tissue to be weaker than it should be.^1−4^ Polymers are promising materials because they show Young’s modulus values similar to those of body tissues, unlike metals, which exceed such values. Nonetheless, polymeric materials require improvements to be mechanically as reliable as metallic devices are, for which they are generally reinforced with fillers to form composite materials.^5,6^

Focusing on resorbable orthopedic medical devices, polylactides (PLA) show an exceptional potential, particularly PLLA.^7,8^ Due to the semicrystalline nature of PLLA, it is possible to tune its mechanical properties by adjusting the crystalline fraction and structure.^9,10^ Specifically, the stiffness of PLLA fits within the elastic modulus range of the trabecular bone, with Young’s moduli of 2–3 and 1–10 GPa, respectively.^11,12^

Furthermore, it is a bioabsorbable polymer with no harmful agents for the body that can be derived upon its degradation. Besides, PLLA is synthesized by the polymerization of lactic acid, which can be obtained from natural resources such as corn, wheat, or rice; therefore, it is a sustainable polymer that is able to develop a circular production and consumption model.^13−15^

Nonetheless, some properties should be improved to suit PLLA for biomedical applications. First, PLLA is composed of O, C, and H elements that have low electron density and specific gravity, making it impracticable to detect by commonly used X-ray imaging techniques.^16^ Second, it is considered to be a brittle polymer at room temperature since high molecular weight PLLA can only show a ductile behavior for a short period of time after solidifying, when its amorphous structure is energetically propitious to permit plastic flow. We acknowledge the ability of PLLA to show ductile behavior, and our work is developed on the basis of understanding, at a segmental level, the mechanism underneath.

**Scheme 1:**
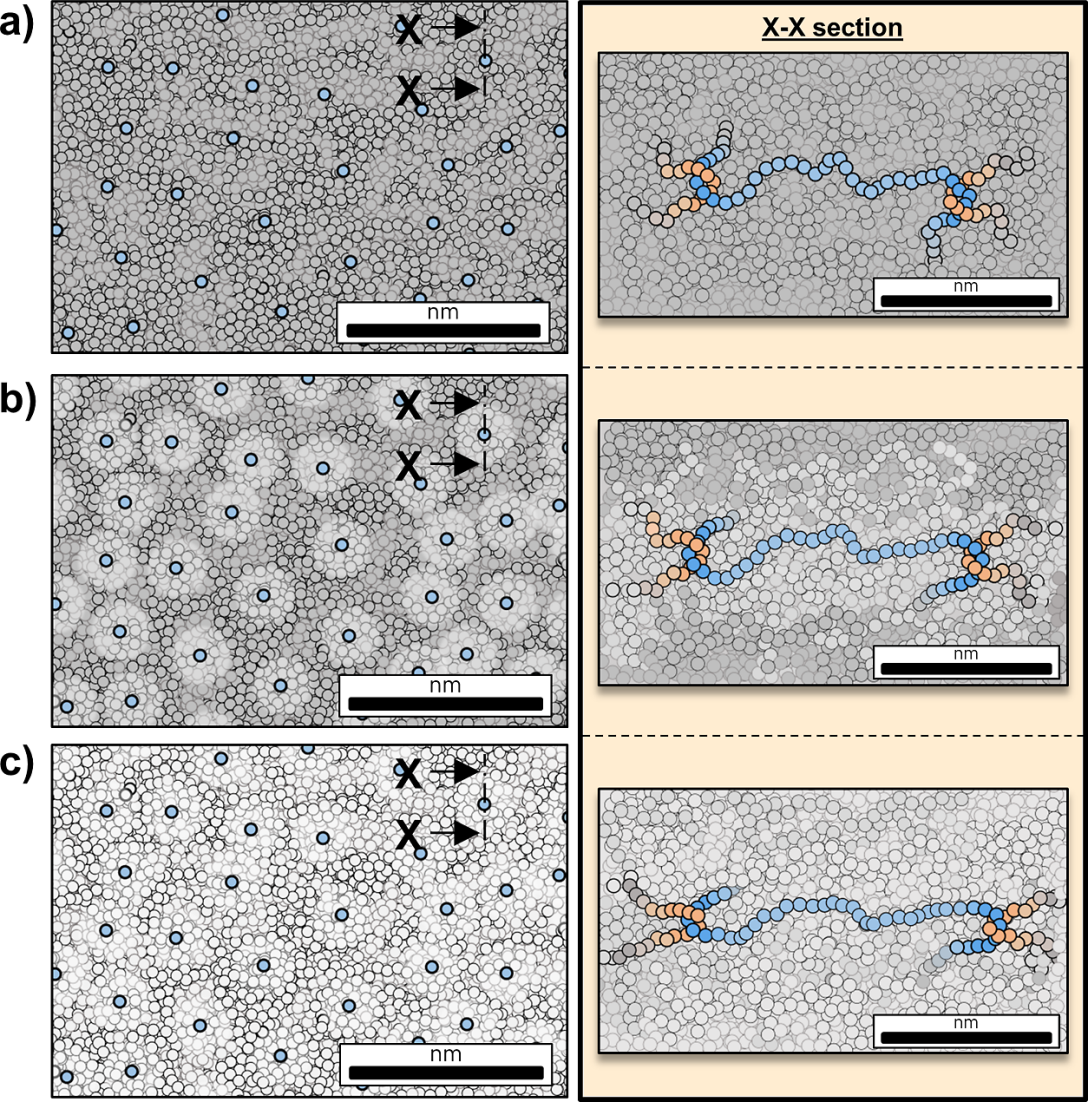
Evolution of the Activation Process on Glassy Polymers upon Extension XX section depicts in blue entwined chains constituting the chain network. (a) Not stretched. (b) Structure partially active, in light gray, with still vitrified mass, dark gray. (c) Whole structure activated and able to yield. Based on the work from Wang et al.^18^

The molecular structure of glassy polymers of high molecular weight, as it is the amorphous fraction of PLLA, is conceived as a hybrid structure. Intersegmental attraction forces between chains comprise the primary structure, and chains entwined in developing junctions between segments, depicted in blue in Scheme 1, constitute the coexisting chain network.^17^ Upon deformation, chains from the chain network are capable of developing enough chain tension to enhance the kinetic thermal energy level of the surrounding segments, as described in Scheme 1 by the lighter coloring of the initially gray landscape. If the increase overcomes the potential activation barrier of the entire primary structure, reaching the condition depicted in Scheme 1c, the amorphous phase undergoes plastic deformation, leading to a ductile behavior of the polymer.^18^

In semicrystalline polymers, the activation of the primary structure is effective on driving the polymer toward a ductile behavior if the group of chains linking crystalline phases with the chain network is strong enough to tear apart the crystalline structure, sustaining plastic flow.^19^

However, the structural rearrangement of chains toward the corresponding lower thermodynamic equilibrium state of the glass known as physical aging,^20−22^ sorely decreases the thermodynamic state of amorphous polymers, making the chain network to activate a smaller fraction of the surrounding primary structure. Under external load, the stress reaches a level where the structure, still with vitrified areas similar to Scheme 1b, is not able to activate and locally fails, leading to the formation of crazes, precursors of cracks, and the brittle breakup of the polymer. The impact of such evolution of the molecular structure in the mechanical properties due to aging has been observed and is still studied in various glassy polymers such as poly(methyl methacrylate), polycarbonate, polystyrene, and amorphous PLLA.^23−27^

In the presence of crystals, this segmental rearrangement shows slower development due to two constraint levels that are given in the amorphous phase, differentiating a mobile amorphous fraction (MAF) and a rigid amorphous fraction (RAF). MAF is given in the bulk of the polymer glass and can be divided into unconstrained MAF, away from crystalline structures, and constrained MAF (cMAF), given in the perimeter of crystalline structures, such as spherulite boundaries. Segments considered RAF are located in highly constrained interlamellar and space-filling intraspherulitic areas, for which a higher glass transition temperature (*T*_g_) has also been reported. Nonetheless, the primary structure is still driven toward lower thermodynamic levels, especially in PLLA, which having the *T*_g_ close to ambient temperature experiences a fast relaxation and early embrittlement.^28−30^

To overcome the challenge posed by the fragility of PLLA, many strategies have been developed, from blending PLLA with softer polymer phases to adding reinforcements to the PLLA matrix. Blending or adding plasticizers to PLLA not only improves greatly the mechanical toughness but also shows enhanced biodegradability when blending is carried out with biodegradable compounds. Even so, in most cases, blending with these softer phases vastly compromises the stiffness of the blend, reducing the Young’s modulus in comparison to neat PLLA. In addition, obtaining good miscibility between compounds tends to be a challenge and can lead to poor mechanical performance of the blend.^31−34^ Nevertheless, in recent years, new blends have been researched in order to preserve the stiffness in ductile PLA blends, such as PLA/poly(ethylene oxide)-*b*-poly(butylene oxide) by McCutcheon et al.^35,36^

Reinforcing the matrix with fillers has been shown to be an effective technique to improve the toughness of PLLA maintaining proper elastic modulus values, yet processing of such composites can be complex due to changes given in the rheological properties of the melt or solution with the addition of the filler. Besides, the resulting distribution of the filler greatly affects the final properties of the composite. Nevertheless, the variety of fillers that can be used as reinforcement broadens the possibilities of adding supplementary properties to the bulk polymer, i.e., not only it is mechanically reinforced but physical, chemical, or biological properties can also be grafted to the composite.^37−39^ Consequently, composites stand out as potential materials for medical devices.

Zhang et al.^40^ studied the toughening and biomineralization-enhancing effect of the addition of octadecylamine-functionalized nanodiamond particles on PLLA for bone tissue engineering, which led to a slight increase in the strain at failure of neat PLLA from 5 to 19%. Muiruri et al.^41^ reinforced PLLA with surface functionalized cellulose nanocrystals to obtain highly improved biodegradability and toughness, reaching strain levels near 250% with minimal loss in stiffness. Similarly, Martinez de Arenaza et al.^42^ reported an impressive improvement on toughness after adding micron-sized barium sulfate (BaSO_4_) inorganic particles to the PLLA matrix. Composites incorporating up to 10 wt % of particles reached an increase of 1600% on the fracture toughness and an increase of 15% on the radiopaque parameter.

These works focus on the improvement of the toughness of PLLA composites without considering the segmental dynamics underneath, which are the origin of their brittle behavior. Ductile behavior within a few days timespan has been achieved by reinforcing PLLA with fillers, yet the ongoing physical aging of the PLLA matrix should be acknowledged for such composite materials.

This being so, we studied the mechanical behavior of PLLA reinforced with different concentrations of BaSO_4_ particles in a long-range aging spectrum, up to 150 days. Using the tensile test as an indicator of mechanical performance and simulating a storage condition aging, hence avoiding any kind of degradation, we aim to remark for the first time the boundaries that such composites entail in regard of physical aging and expose the underneath behavior of the polymer segments based on the most recent segmental dynamics theories.

## 2 Experimental Methods

### 2.1 Material

Commercial PLLA of molecular weight (*M*_w_) of 175,000 g/mol and l isomer content higher than 99% obtained from Total Corbion (Luminy PLA L175) was used in this study. Barium sulfate (BaSO_4_) particles were supplied by Sigma-Aldrich. Particle size distribution in volume and number of BaSO_4_ was characterized by using a laser scattering particle size distribution analyzer (HORIBA LA-350). Measured mean particle size (equivalent diameter) in number and volume models are 1.45 and 2.05 μm, respectively. Scanning electron microscopy (SEM) imaging detecting backscattered electrons (BSE) shows an angular particle morphology with intermediate sphericity. Elemental analysis from energy-dispersive X-ray spectroscopy in the particles confirms the presence of Ba, S, and O. Particle size distribution and morphology are both shown in Figure S1 in the Supporting Information.

### 2.2 Sample Preparation

Tensile test samples with a cross section of 1 × 4 mm^2^ were prepared by melt blending using a vertical DSM Xplore model 5 minimixer and injecting the melt into a dumbbell-shaped mold by a Micro Injection Molding Machine of 10 cm^3^. Vacuum-dried (24 h) PLLA pellets were fed together with ascending BaSO_4_ particle quantities of 0.5, 1, 5, and 10 wt %, labeled as PLA_0.5, PLA_1, PLA_5, and PLA_10, respectively. Materials were mixed at 200 °C, 150 rpm, and torque between 10 and 13 N m for 1.5 min to ensure good particle dispersion. Injection was carried out at 16 bar pressure with the mold heated at 45 °C. Samples were broken in half after cooling in a liquid nitrogen bath to check the dispersion of particles in the matrix. A uniform particle dispersion was observed in the samples, as seen in the SEM-BSE image in Figure S1c for PLA_10. Unfilled specimens, labeled PLA_*N*, were processed under the same conditions. The aging process took place in a controlled ambient temperature for all samples by storing them at 21 ± 2 °C and 50 ± 5% relative humidity, simulating a storage aging condition.

### 2.3 Measurements

Thermal transitions and aging were determined by means of the differential scanning calorimetry (DSC) technique on a TA Instruments DSC 200. Samples extracted from tensile test specimens at progressive aging times were hermetically sealed in aluminum pans and analyzed from −20 to 220 °C at a rate of 20 °C/min.

Crystal fraction (*X*_c_) was measured by eq 1, where Δ*H*_m_, Δ*H*_c1_, and Δ*H*_c2_ are the enthalpy of melting, cold crystallization enthalpy, and high-temperature crystallization enthalpy, respectively. We used an enthalpy of melting of perfect α PLLA crystals of Δ*H*_m_^0^ = 106 J/g selected from the bibliography.^43^
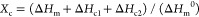
1

The heterogeneous crystallinity of the samples and DSC scans hampers the obtention of reference curves that are correctly superposable with target curves due to the differences given in the change of the specific heat capacity (Δ*C*_p_) upon the glass transition. Therefore, we are not able to properly obtain the relaxation enthalpy (Δ*H*_r_), as it is commonly measured in polymer glasses.^44,45^ This being so, Δ*H*_r_ values were obtained by measuring the area of the endothermic relaxation peak from a horizontal baseline located on the heat flow plateau above *T*_g_ as a way to monitor the additional heat needed to overcome the glass transition due to physical aging. We define *T*_g_ as the temperature at which the highest slope of the thermogram is given within the glass transition. Both methods are visually described in Figure S2a,b in the Supporting Information, respectively. Values are fitted to the number of PLLAs present in each composite. Relaxation rate (β_H_) is obtained from the slope of the linear regression (*R* > 0.98) applied to the Δ*H*_r_ values versus aging time in decades.

Tensile tests were performed in an Instron 5565 at a crosshead displacement of 5 mm/min, initial length *L*_0_ = 40 mm, and progressive aging times (5, 10, 20, 30, and 150 days). Temperature and relative humidity are maintained at 21 ± 2 °C and 50 ± 5% throughout the whole experiment. Debonding stress is taken from the point where the slope change is given in the elastic region of stress–strain curves, calculated from the first derivative of the curve as shown in Figure S4 in the Supporting Information.

Dispersion of particles and microscopic analysis of the sample surfaces were carried out using SEM in a Schottky-type microscope model JEOL JSM-7000F. An acceleration voltage of 10 kV with a beam current intensity of 0.025 nA was used with a working distance between 5 and 20 mm, depending on the needs of each sample.

X-ray powder diffraction patterns were collected by using a Philips X’pert PRO automatic diffractometer operating at 40 kV and 40 mA, in theta–theta configuration, secondary monochromator with Cu Kα radiation (λ = 1.5418 Å) and a PIXcel solid state detector (active length in 2θ = 3.347°). 1° fixed soller and divergence slit giving a constant volume of sample illumination were used. The irradiated area of the sample was approximately 1 cm.

X-ray microdiffraction patterns were collected by using a Bruker D8 Discover diffractometer equipped with a Cr Twist tube, V filter (λ = 2.2911 Å), PolyCap (1 μ single crystal cylinders) system for parallel beam generation (divergence of 0.25°), and a 1-D LynxEye detector (active length in 2θ 2.7°). The samples were mounted on an Eulerian cradle with automatic controlled X–Y–Z stage. The irradiated area was collimated to 1 mm.

Texture evaluation was measured in the same piece of equipment and cradle. Data were collected for the main reflection at 24.3° in 2θ using a fixed mode and time per orientation of 40 s. The data collection in thinned mode with 5° of δ was measured for full circle 0–360 incr. 5° in Phi(ϕ) and 0–70 incr. 5° in Psi(ψ) range giving 621 total orientations.

## 3 Results and Discussion

### 3.1 Aging on Neat PLLA

First, we studied the capability of PLLA to show ductile behavior and the aging time needed to observe a brittle rupture of the samples. PLA_*N* samples were tensile tested 1 to 5 days after processing for this purpose.

**Figure 1:**
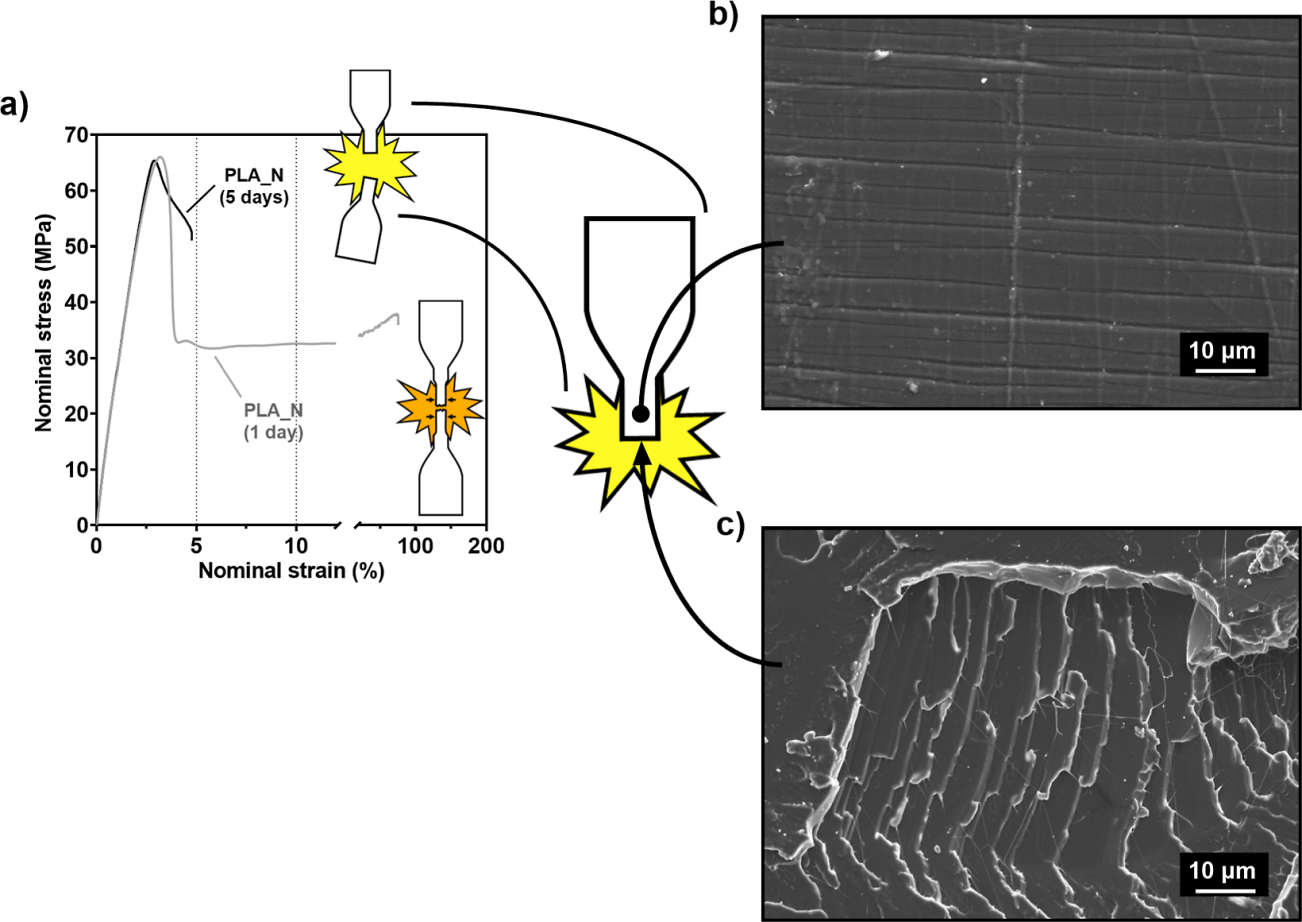
(a) Stress–strain curves of uniaxial tensile tests of PLA_*N* aged for 5 days (black) and PLA_*N* aged for 24 h after processing (gray). Brittle and ductile breakups are represented within the graph as yellow and orange flashes, respectively. (b) SEM image of the crazed side face of the sample. (c) SEM image of the break surface after brittle failure.

Figure 1a collates the stress–strain curves of PLA_*N* aged for 1 day (gray line) and 5 days (black line) in which a change in the mechanical behavior is observed. Samples aged for 1 day exhibited shear yielding and necking, coincident with the yield point of σ_*y*_ = 65 MPa and the stress drop near ε = 4%, characteristic of a ductile behavior. The energy absorbed throughout stretching had effectively activated the primary structure of PLLA, and the samples reached break-up strain values close to ε_r_ = 100%. The ductile behavior is characterized with an orange color in this work, as depicted in the miniature of the sample in the figure. After 5 days of physical aging, PLA_*N* showed the characteristic stress–strain curve commonly attributed to PLLA, showing peak elongation values of ε_r_ = 5%. Samples did not develop necking and experienced an abrupt failure; hence, they are considered brittle. Brittle breakup is characterized with a yellow color in this work, as depicted in the miniature of the sample in the figure. A 5 day time span ensured the embrittlement of the injection-molded samples used in this study.

Comparing the stress levels of the curves after the yield point until necking or brittle failure takes place, brittle samples sustained stress values higher than those of ductile samples. This denotes a higher energy absorption for the rupture of brittle samples than that needed for the necking of ductile samples. PLA_*N* aged for 1 day absorbed a mean value of 1.68 J/cm^3^ for necking to take place, while after 5 aging days, energy absorption values in brittle samples increased up to 2.02 J/cm^3^.

Figure 1b,c shows the SEM images of the side surface near the rupture and the cross breakup section of brittle PLA_*N* samples, respectively. A noticeable presence of crazes can be observed on the side surface of the sample, seen as horizontal stripes in Figure 1b. Cross breakup section image shows flat-leveled surfaces, indicating that the structure has not developed plastic deformation and the rupture propagated throughout the material in a brittle way.

The primary structure in PLA_*N* samples aged for 5 days was unable to activate and locally failed, leading to the generation of crazes. The apparent yielding observed in the stress–strain curve is achieved by a massive generation of crazes, denoted as craze-yielding,^46^ which permits further elongation without the overall activation of the primary structure. In the absence of any overall activation, the structure kept absorbing energy as it stretched until crazes developed into cracks that quickly propagated, leading to the observed abrupt brittle failure.

**Figure 2:**
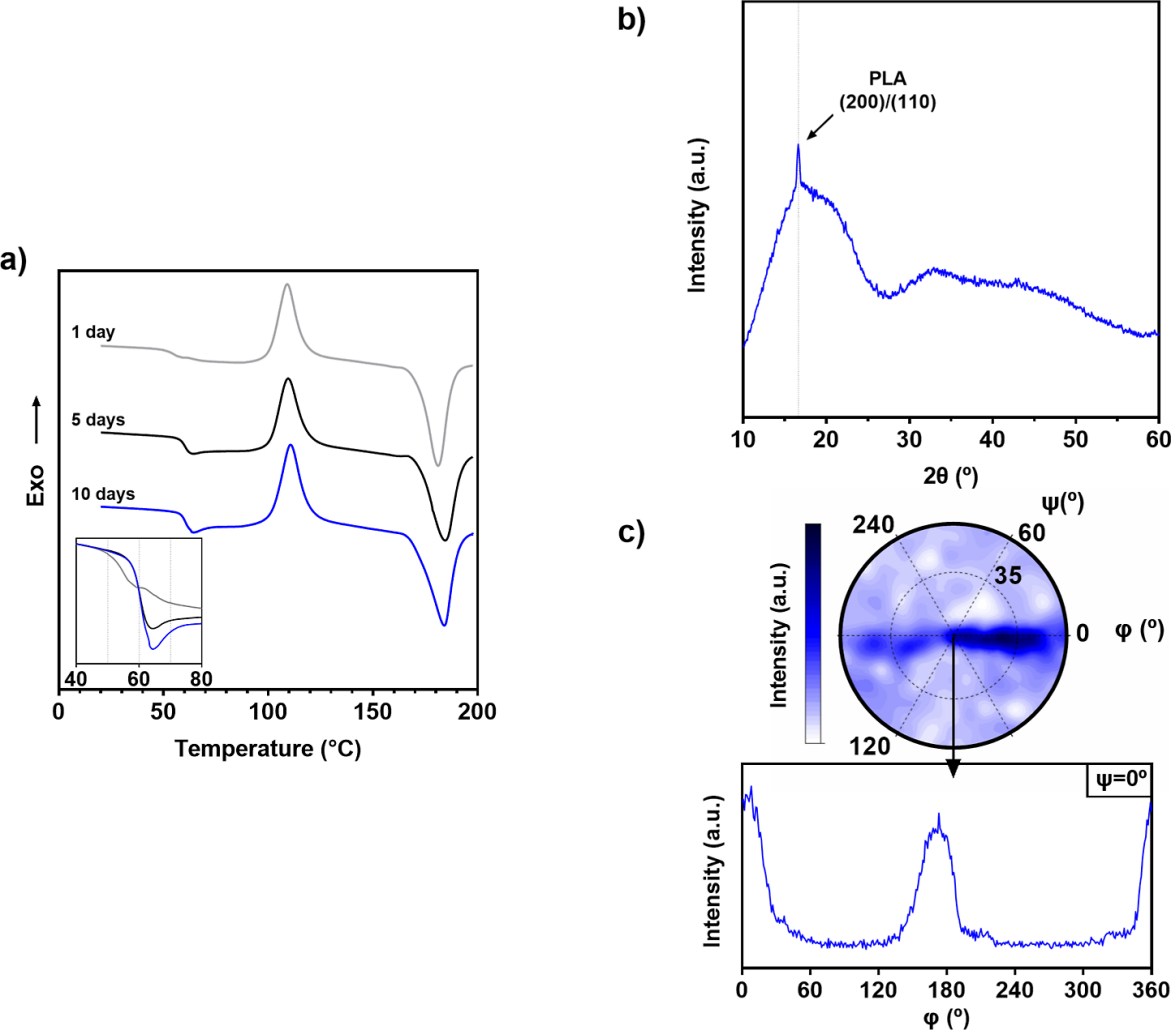
(a) DSC curves of PLA_*N* aged for 1 (gray), 5 (black), and 10 days (blue) (check the online version for an optimal view). Curves are plotted superposed along the glass transition regime in the bottom left corner detail. (b) X-ray diffraction curve of PLA_*N* aged for 10 days. (c) Contour plot of the texture analysis of PLA_*N* crystal in 10 days aged sample. Phi scan of the nontilted sample is given in the graph underneath.

In order to relate the observed mechanical behavior of PLA_*N* samples with the condition of their chain structure, the evolution of chain relaxation and the crystalline state of the polymer must be characterized. We performed DSC measurements of samples aged for 1, 5, and even 10 days after processing to characterize the thermal properties of the samples and X-ray diffraction (XRD) measurements to analyze the crystalline structure developed during injection molding. Results are shown in Figure 2, where curves from DSC measurements are shown on the left side (a) and XRD data is depicted on the right (b,c).

Focusing on DSC results, all three curves show similar cold crystallization and melting peaks in both temperature and shape, yet changes can be observed in the signal during the glass transition. The detail on the bottom left of the graph overlays the three curves in the glass transition regime. Samples measured 1 day after processing (gray line) show a broad transition with no relaxation enthalpy, indicating no physical aging for these samples. Nevertheless, after 5 aging days (black line), this relaxation peak is visible, and for samples aged for 10 days (blue line), it is more pronounced.

Values of thermal transitions and the crystal fraction of the DSC measurements are summarized in Table 1. The effect of aging is observed in both the increase in *T*_g_ and the value of Δ*H*_r_. The glass transition was initially located at 55.5 °C and reached 60.4 °C after 5 days of aging. Further aging did not affect *T*_g_, but Δ*H*_r_ did increase between 5 and 10 days of aging, from 1.3 to 3.5 J/g. This indicates that PLA_*N* kept bearing physical aging even after embrittlement at 5 days, when the thermodynamic state of the primary structure was already too low to reach activation.

**Table 1:** DSC Results of PLA_*N* Aged for 1, 5, and 10 Days

sample	aging (days)	*T*_g_ (°C)	Δ*H*_r_ (J/g)	Δ*H*_ci_ (J/g)	Δ*H*_m_ (J/g)	*X*_c_ (%)
PLA_*N*	1	55.5	0.0	43.3	48.5	4.9
PLA_*N*	5	60.4	1.3	37.3	42.7	5.1
PLA_*N*	10	60.5	3.5	40.5	44.6	3.8

The value of crystal fraction measured from thermal transitions shows a low value of crystallinity for the samples, near the 5%. These values are consistent with the results obtained from XRD measurements.

Concerning the wide-angle X-ray scattering (WAXS) diffractogram in Figure 2b, an amorphous halo with a predominant crest at lower 2θ values can be observed. This denotes an oriented glassy structure in which a single crystallinity peak at 2θ = 16.6° can be distinguished, corresponding to the (200)/(110) plane of α form PLLA crystals.^47^ Additional measurements were done with 1 and 5 days aged samples, but no change in crystalline phases was observed due to aging. Figure S3 shows the single crystalline peak at 2θ = 16.6° for both aging stages, the same observed after 10 aging days. Regarding texture analysis in Figure 2c, crystals are oriented toward the injection direction and slightly tilted, as can be observed in the displaced signal of the contour plot.

The presence of oriented crystals has been reported to enhance stiffness and toughness in PLLA,^48^ besides constraining the segmental relaxation of the amorphous structure, which explains how PLLA endured 5 days before showing a brittle behavior. Nevertheless, DSC results depict that physical aging keeps increasing after embrittlement, which implies that the primary structure of PLLA is becoming even more difficult to activate. Consequently, we find it necessary to analyze the mechanical behavior of PLLA and BaSO_4_ composites with physical aging beyond 5 days.

### 3.2 Aging in PLLA/BaSO_4_ Composites

**Figure 3:**
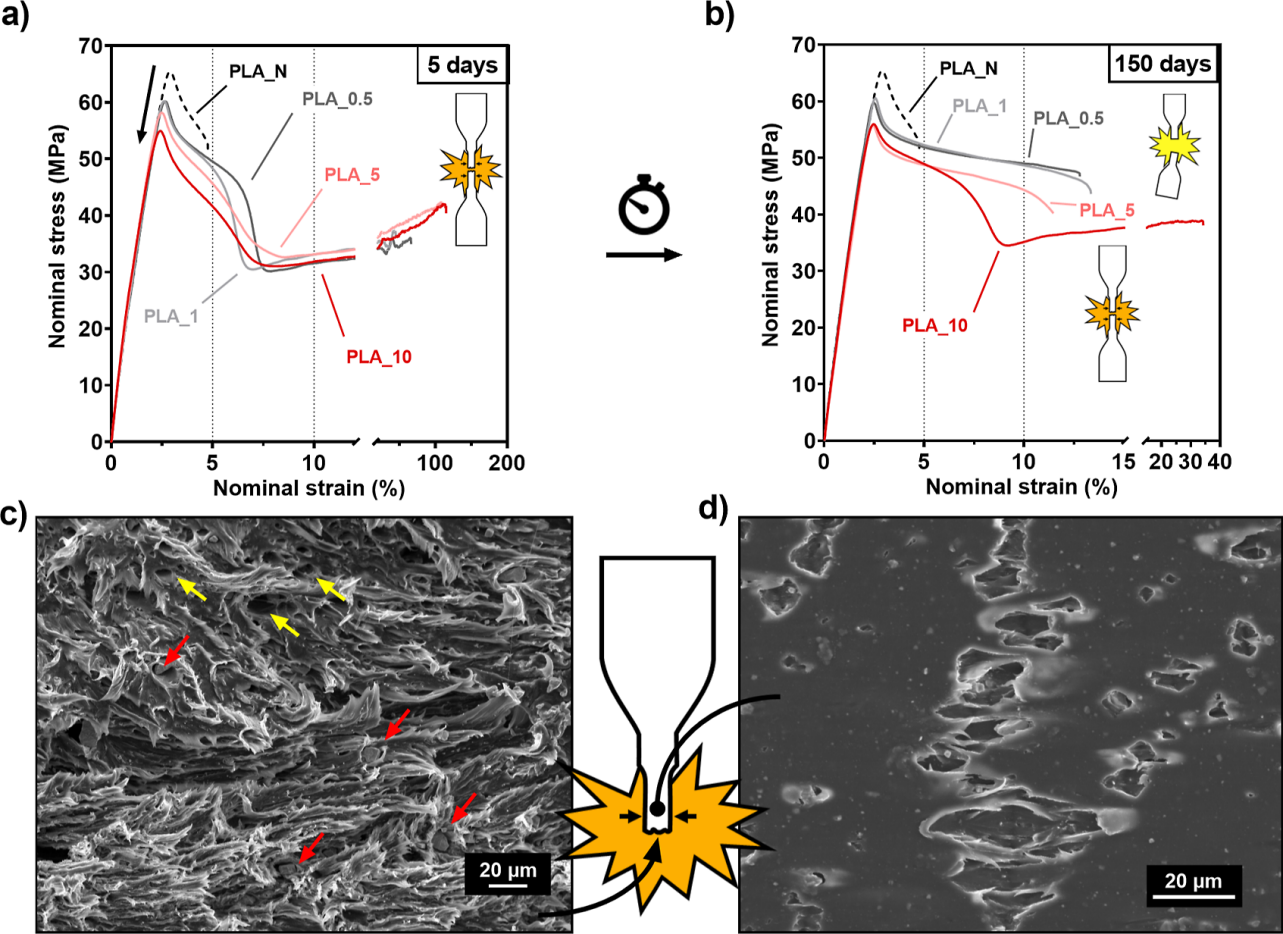
(a) Stress–strain curves of uniaxial tensile tests of composite samples aged for 5 days and 5 days aged PLA_*N* as reference. (b) Stress–strain curves of uniaxial tensile tests of composite samples aged for 150 days and 5 days aged PLA_*N* as reference. (c) SEM image of the break surface of ductile PLA_10, red arrows show the position of particles, while yellow arrows point out cavities left by debonded particles. (d) SEM image of the side face near the rupture of ductile PLA_10.

Mechanical behavior and properties of the composite samples were also studied by tensile testing. Figure 3 shows the stress strain curves for PLA_0.5, PLA_1, PLA_5, and PLA_10 aged for 5 days (a) and 5 months (b), together with SEM images of the rupture face (c) and the side surface of the sample close to it (d). Tensile test of PLA_*N* of 5 aging days is depicted as a reference in a black dashed line in both graphs.

Focusing on Figure 3a, the effect of particle addition is first noticeable in the elastic region of the curve. The Young’s modulus increases with the addition of particles. PLA_*N* samples showed stiffness values of 2.6 GPa, while with the addition of 10 wt % of particles, it reached 3.1 GPa. Nonetheless, a clear loss of yield strength can be noted, as indicated by the black arrow, which is related to the debonding effect. The yield stress for PLA_*N* is 65 and 60 MPa for PLA_0.5, decreasing down to 55 MPa for PLA_10. Debonding refers to the physical separation of the particle–matrix interface and makes the linear-elastic behavior to be lost at lower stress levels,^49^ known as debonding stress. In agreement with this premise, debonding data obtained from tensile tests depicted in Figure S4 show a debonding stress decrease of 7.6% when enhancing from 0.5 wt % of particles to 10 wt %. The debonding phenomenon is related to the interphase between particle and matrix; hence, it is not found to be time-sensitive. Any variance observed in the values is expected to come from the wide range of particle sizes and the effect that local crazing mechanisms might have within each individual sample, especially in those samples that showed a brittle behavior.

The intended toughening effect was achieved with the addition of BaSO_4_ particles since 5 days after injection molding, all the composite samples showed a ductile behavior and an enhanced toughness. Composites reached high plastic deformation values, showing higher strains at break as the added particle amount increased. PLA_0.5 and PLA_1 elongated to values around ε_r_ = 80% of their initial length, whereas PLA_5 and PLA_10 showed strain values up to ε_r_ = 120%. Remarkably, necking takes place with the stress drop right before the stress plateau initiation, at higher strains than that experienced in ductile PLA_*N*. In comparison, stress–strain curves of 1 day aged composites in Figure S5 in the Supporting Information show that samples undergo necking at strains similar to those of ductile PLA_*N*, which indicates that the effect of aging is already noticeable after 5 aging days. Moreover, higher particle quantities decrease the debonding stress of the composites as seen previously, but these fresh samples show values closer to the yield stress of ductile PLA_*N*, denoting that particle–matrix adhesion is weakened in early stages of physical aging.

In Figure 3c,d, SEM images reveal characteristic cavities formed by particle debonding and expose the plastic failure mechanism underneath the ductile behavior of composite samples. In the rupture section in Figure 3c, a wide particle size range can be noted as well as their homogeneous distribution within the matrix, distinctly located inside the voids (red arrows). Some hollow cavities (yellow arrows) are also left due to the loss of particles after the break. Figure S6a in the Supporting Information shows the same SEM-BSE side by side with the original image in which the particles are easily differentiated from the matrix. The PLLA matrix is found to be totally distorted full of thread-like shapes, which indicates that the structure has been activated and the plastic flow has been induced, hence the observed ductile behavior.

Moreover, Figure 3d shows the morphology of the side surface of the sample that in contrast to Figure 1b has no presence of the crazes seen in PLA_*N*. Instead, interconnected cavities developed from particle debonding are observed. These cavities meet a similar distribution on the surface of the sample as the massive crazing previously seen; however, their internal structure has undergone plastic deformation, and propagation is more energy-consuming and stable than in regular crazing, which also explains the improvement in toughness.

Finally, it is concluded from the tensile test results in Figure 3b that the composite samples aged for 150 days are also prone to show brittle behavior due to segmental relaxation. From all composite systems, only the curve corresponding to PLA_10 shows a ductile behavior. The degree at which ductile behavior is lost is strongly linked to the amount of particles added to the matrix. Table 2 summarizes the aging time needed for each composite system to exhibit brittle behavior. PLA_0.5 and PLA_1 showed a brittle behavior 20 days after injection molding, whereas PLA_5 endured over a month of retaining ductility. On top of that, being PLA_10 the only composite that remained ductile all over the 150 days studied in this work, it also experienced a reduction on the elongation at break down to ε_r_ = 35% at the last aging stage. Comparing the break surface of PLA_10 seen in Figure 3c to the one from already embrittled PLA_5 in Figure S6b, the latter shows a plain break surface, and even if it shows rounded edges in the structure, more than in brittle PLA_*N* seen in Figure 1c, there are no signs of an overall plastic deformation of the matrix.

**Table 2:** Summary of the Durability of the Ductile Behavior in Composite Samples^a^

	aging(days)				
sample	5	10	20	30	150
PLA_0.5	●	●	X	X	X
PLA_1	●	●	X	X	X
PLA_5	●	●	●	●	X
PLA_10	●	●	●	●	●

a The dot refers to a ductile behavior, while the cross means embrittlement.

It is remarkable that both particle addition and physical aging affect the behavior of the samples after the yield point in the stress–strain curve. As mentioned, the shape of the curves of composite samples in Figure 3a is wider compared to the samples aged for 1 day, i.e., the plateau stress stage is achieved at higher strain levels. Even further, these values keep increasing as aging time is prolonged. In Figure 3b, PLA_10 necks at strains close to 10% and for the rest of the composites, strain values at break largely increased, surpassing the 10% in all cases. This behavior fits in agreement with the SEM results, showing that particle debonding effectively improves the deformability of the composite reaching a higher deformation strain.

**Figure 4:**
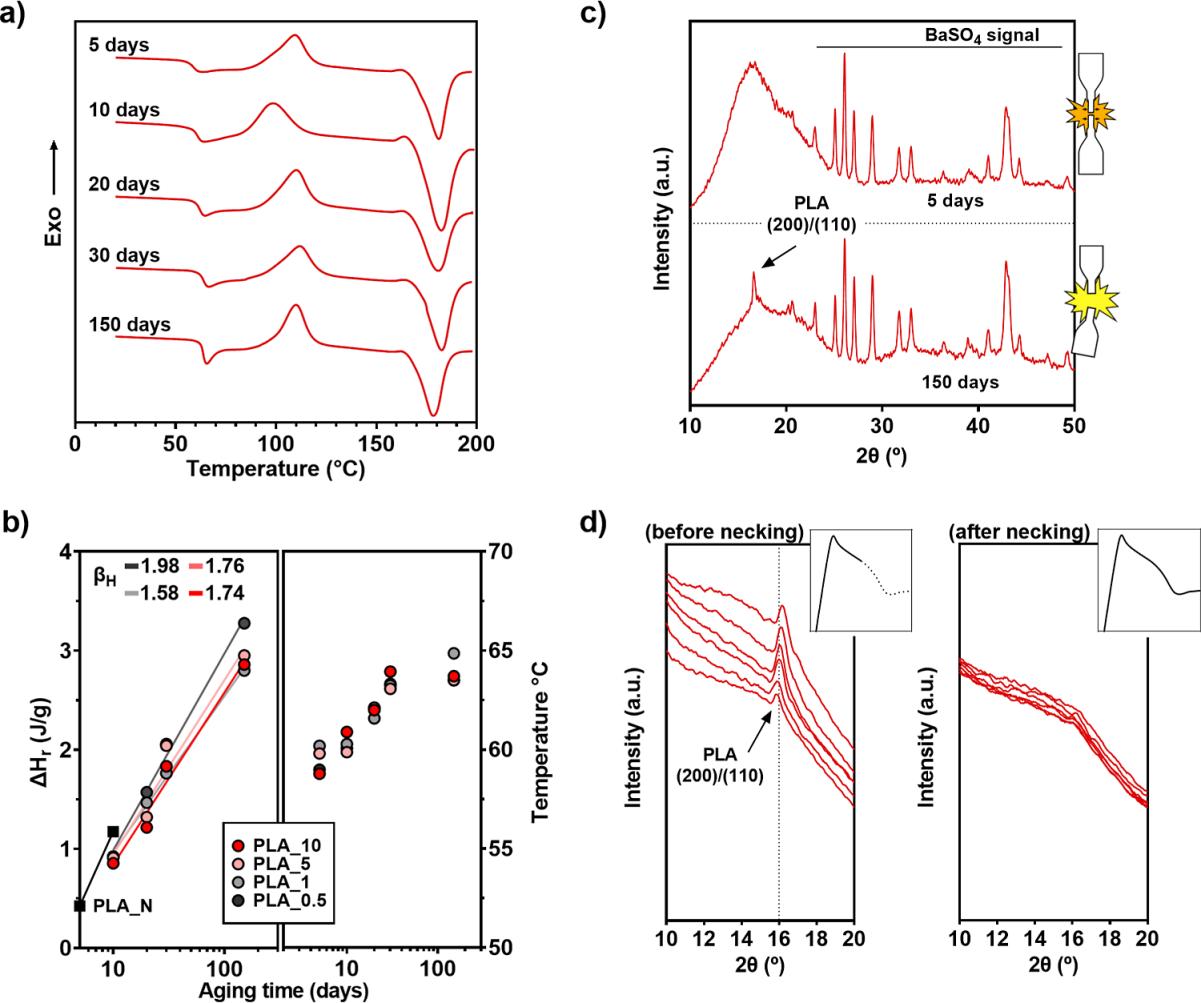
(a) DSC curves of PLA_10 aged for 5, 10, 20, 30, and 150 days. (b) Evolution of enthalpy relaxation values and rates in [J/g·dec] (left) and *T*_g_ values (right) of composite samples as a function of aging time. Enthalpy relaxation values of PLA_*N* are shown as black square icons. (c) X-ray diffraction curves of tensile-tested PLA_5 samples, 5 days aged (up) and 150 days aged (down). (d) Curves from microWASX scan of stretched PLA_5 before necking (left) and after necking (right), 6 measurements along the sample axis.

Similar to PLA_*N*, the segmental structure of the PLLA matrix in the composites was studied by means of DSC and XRD. Figure 4 shows data obtained from DSC scans in the left column (a,b) and WAXS measurements in the right column (c,d).

Regarding thermal properties, Figure 4a contains DSC curves of every aging stage of PLA_10 and Figure 4b displays the values of Δ*H*_r_ and *T*_g_ of every system at each aging stage. All composites showed a similar thermogram evolution in time, so we chose to graph the curves corresponding to PLA_10 in order to simplify the figure and portray in a clearer way the aging behavior of these samples. Additionally, a characteristic effect of the addition of particles is the broadening of the cold crystallization peak in contrast to PLA_*N*, hence making impossible the reading of Δ*H*_r_ for most samples at 5 days of aging. Therefore, these values are not plotted in Figure 4b.

The *T*_g_ of PLA_10 raised from 59 °C up to 64 °C, and similar values were measured for the rest of the composites, yet PLA_1 outstands after 150 days of aging with a slightly higher *T*_g_. In addition to an increased *T*_g_, the Δ*H*_r_ is enhanced to a more severe extent. All composites share a matching upward trend and Δ*H*_r_ values, near 1 J/g at 10 aging days, lower than that of PLA_*N*, and almost reaching 3 J/g after 150 days. A slight difference is taken for 150 days between a content of 0.5% particles and the other ones, relaxation enthalpy of PLA_0.5 stands at 3.2 J/g at this point. Calculated enthalpy relaxation rates show almost the same value for PLA_5 and PLA_10, 1.74 and 1.76 J/g·dec, respectively, and a lower rate of 1.58 J/g·dec for PLA_1. As expected from the higher Δ*H*_r_ from PLA_0.5, the system shows the highest β_H_ of 1.98 J/g·dec among all composites. As a reference, measured β_H_ of PLA_*N* between 5 and 10 aging days is 2.49 J/g·dec. PLA_*N* showing the highest β_H_ from all suggests that the presence of particles hinders segmental movement, and similarly, PLA_1 stands out as the most effective one in reducing the relaxation rate. Nonetheless, relaxation enthalpy values of further aging stages of PLA_*N* would be needed to properly fit the real β_H_ values of the bulk polymer. On top of that, composites show varying yet similar Δ*H*_r_ values throughout the aging process, and a slight variation in the rate is negligible compared to the vast difference exhibited in the mechanical performance, e.g., PLA_5 and PLA_10 showing a ductile behavior after 30 aging days in contrast to already embrittled PLA_1 with a lower relaxation enthalpy. Therefore, we find the increment of the relaxation enthalpy of the PLLA matrix during the aging process to be similar for all composites, which leads us to address the effect of BaSO_4_ particles toward PLLA segments.

A severe constrain of the segments diminishes the change of the specific heat capacity (Δ*C*_p_) upon the glass transition of the polymer, as seen in PLLA samples with a high amount of RAF.^50^ In our samples, the measured Δ*C*_p_ in the glass transition is dependent on the crystallinity of each sample and has no relation with the amount of particles in the composite, as seen in Figure S7 in the Supporting Information, where all composite systems are scattered over all the Δ*C*_p_ range. Therefore, we confirm that the highly constrained segment fraction in the composites is derived only from RAF. Moreover, previously mentioned similar values in the *T*_g_ of samples also depict no change in cMAF^51^ between composites, indicating that the interaction between particles and matrix barely shows any macroscopic evidence of segmental constraint. Altogether, in contrast to the time extended mechanical toughness achieved in the work, the addition of a larger quantity of particles has shown to have little impact on the measured parameters of segmental constraint and relaxation. This means that the degree of segmental relaxation within the samples when tensile testing was the same in each aging stage regardless of the quantity of particles added, which confirms that a higher particle addition enables a ductile behavior of the samples at longer times after processing.

These results seem to differ with studies regarding polymeric nanocomposite systems. A strong interaction between fillers and matrix, along with diminishing the segmental relaxation rate, has been reported for composites with nanosized fillers (<100 nm).^52−54^ However, the microparticles used in our study show much larger dimensions (∼2 μm), meaning that they have less surface area to volume ratio to interact with the matrix and a wider spacing between particles; thus, considerably more polymer bulk is unaffected by particle presence.

Getting back to the enlarged stress–strain curves observed in Figure 3b, it is of great interest to analyze the state of the primary structure in the samples throughout the tensile test, i.e., whether it has been activated or not. As mentioned, crystalline structures can be dismantled only if the primary structure has been effectively activated; therefore, XRD analysis can be carried out to confirm if crystals are gone due to activation of the amorphous phase. With this purpose, the WAXS measurements were done in ductile and brittle samples.

Figure 4c shows diffractograms of tensile-tested PLA_5 samples, one that exhibited ductile behavior at an early aging stage (upper graph) and another that already embrittled after 150 days of aging (lower graph). Both graphs show unaltered peaks of BaSO_4_ crystals above 2θ > 20°, but a change can be observed in the peak characteristic of PLLA crystals. The brittle sample still displays the peak at 2θ = 16.6° corresponding to the (200)/(110) plane of α form PLLA crystal, as PLA_*N* did, but no sign of PLLA crystallinity can be found in the diffractogram of the ductile sample. Moreover, a more pronounced crest of the amorphous halo is shown in the latter one, a result of a higher orientation of chains due to the imposed plastic deformation.

From these results, we can confirm that the primary structure of the PLLA used for this study is capable of tearing the crystalline structure and showing a ductile behavior once it is activated. Consequently, if the brittle samples still display crystalline phases, we can deduct that in those cases, the primary structure has failed prior to activation, i.e., activation is not achieved in the brittle samples.

This being so, we aimed to establish the point where crystals disappeared in the tensile test in order to determine the moment of activation of the primary structure. Two samples of PLA_5 aged less than 5 days were partially tensile tested for this purpose. The stretching of the first sample was stopped after the yield point of the curve was reached, but before necking could take place. The second sample was let to develop necking, and the stretching was stopped right after the stress drop, when the neck propagation began. Six points with 5 mm spacing along the axis of each sample coincident with each curve in the graph were analyzed by means of micro-WAXS in the affected area, either whitened or necked. Results are shown in Figure 4d left and right, respectively. A detail of the stress–strain curve indicating the stop of the stretching is added in each graph.

Crystallinity peaks, now shifted to higher angles due to the change from Cu radiation (λ = 1.5418 Å) to Cr radiation (λ = 2.2911 Å), are present in the sample before necking but not after necking happened. Accordingly, we now can delimit the activation of the primary structure to the stress drop of the stress–strain curve, which from now on will be referred to as the activation point.

### 3.3 Macroscopical Approach to Segmental Dynamics

All of the analysis carried out up to this point has led to the understanding that physical aging, hindering the activation of the primary structure, takes the activation point to higher strains. Remarkably, the presence of particles in the matrix enables the material to deform way further than neat PLLA could, either achieving activation or failing seeking it. This shift on strain can also be addressed as an increase on the absorbed energy throughout the stretching of the sample, i.e., it is possible to relate the change in the energy absorbed by the samples in the tensile tests with the activation process of the primary structure. Therefore, the state of the primary structure can be characterized by measuring a macroscopic parameter.

**Figure 5:**
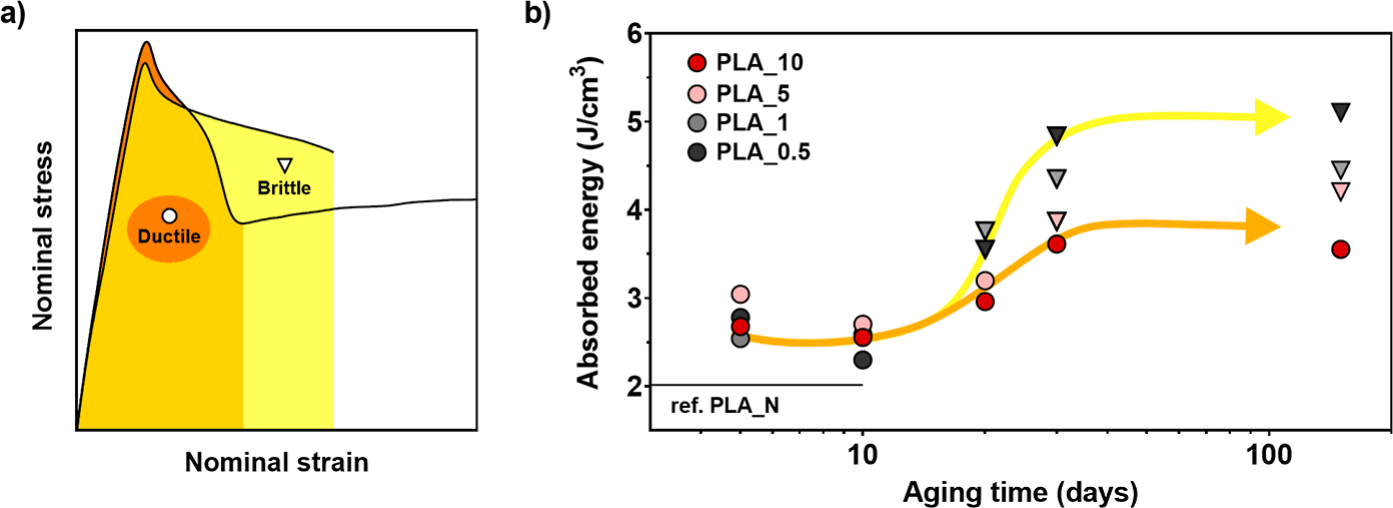
(a) Example of how absorbed energy is calculated for brittle samples, yellow fill and triangle symbol (▼), and ductile samples, orange fill and circle symbol (●). (b) Measured absorbed energy from uniaxial extension by the composite samples as a function of aging time. The value corresponding to brittle PLA_*N* is plotted as a solid black line as a reference.

Based on this premise, we measured the energy absorbed by the samples in the activation process for each tensile test, and the evolution of these values in time is depicted in Figure 5b. For ductile samples, activation energy was obtained by integrating the area behind the curve between the initiation of the tensile test and the activation point, whereas for brittle samples, the whole curve was integrated as the sample was unsuccessfully trying to reach activation. This is graphically described in Figure 5a. Ductile samples are characterized by circles, and brittle samples by triangles in the graph for a better understanding.

First, results show increased energy consumption for the composite samples compared to the values obtained from brittle PLA_*N* in the 5 to 10 aging days range. In the very beginning of the aging process, when all of the composite samples showed ductile behavior, absorption values did not vary much among them. However, samples developed unevenly absorbed energy increments upon physical aging.

After 20 days of aging, the samples with low particle quantity began to show a brittle behavior, and their energy absorption increased, deviating in trend, as depicted with a yellow arrow in the figure, from the samples that still remained ductile. PLA_0.5 and PLA_1 show energy absorption levels getting close to 4 J/cm^3^, behaving in a brittle manner; whereas PLA_5 and PLA_10 remain ductile and absorb 3 J/cm^3^, less than PLA_0.5 and PLA_1. After 30 aging days, brittle PLA_0.5 and PLA_1 already at high energy absorption levels show values of 4.9 J/cm^3^ and 4.4 J/cm^3^, respectively. Values, which will be retained for the completion of the study. PLA_5 unable to show a ductile behavior after 30 days still presents absorption values similar to PLA_10, but get higher after 150 days getting close to PLA_1 with 4.2 J/cm^3^. Above all, PLA_10 absorbed considerably less energy after 150 days of aging, 3.5 J/cm^3^, and showed ductile behavior throughout the study.

**Scheme 2:**
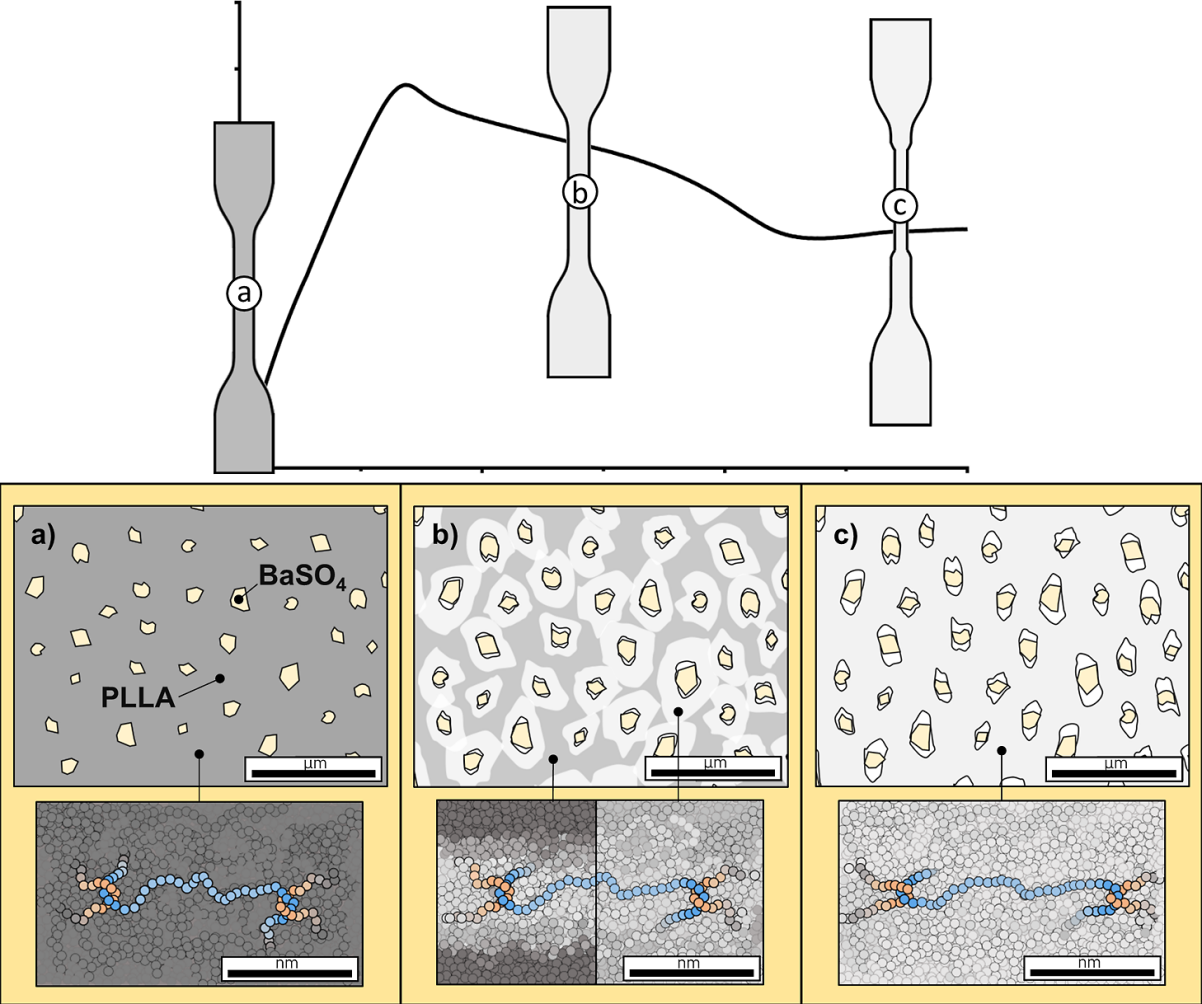
Illustration of the Toughening Mechanism of an Aged Composite Sample with High Particle Quantity (a) Initial state of non-stretched vitreous primary structure (dark gray). (b) Stretched sample after debonding took place. Lighter gray areas depict enhanced energy levels. (c) Necked sample showing a totally activated primary structure.

These results show that as the energy needed to activate the primary structure increases, the mechanical energy absorbed by the samples becomes more sensitive to the number of particles in the matrix. Essentially, a higher quantity of particles activates a higher fraction of the matrix, leading to an earlier overall activation of the structure. Brittle samples keep absorbing energy with no overall activation until imminent failure takes place. This effect is depicted in Scheme 2, where the mechanism that governs the mechanical behavior observed in this study is described. The image relates the segmental structure and microscopic section of a ductile composite sample with three subsequent stages of a tensile test: (a) before loading, (b) after yield point, and (c) after necking.

Upon enough elongation, debonding initiates localized strains in the particles’ surroundings, easing the activation (light gray) of the corresponding primary structure. This phenomenon leads to the state depicted in Scheme 2b, where the fraction of the matrix located near the debonded particle has overcome the activation barrier while the intermediate area still remains vitrified (dark gray). At this point, deformation is permitted by the localized plastic flow of the active fractions and the development of the interconnected cavities derived from debonding, as depicted in Figure 3d.

If the physical aging of the matrix is not that severe, activation of the primary structure will extend through the whole matrix at lower strains. Similarly, if the number of uniformly dispersed particles in the composite is high enough, activated areas will be closer from each other, and less energy will be needed for them to span the whole section. Either way, both result on an overall activation, necking, and consequent ductile behavior, as depicted in Scheme 2c. This refers to the behavior observed in all the composite samples at 5 aging days and only for PLA_10 after 150 aging days.

Conversely, a sorely aged composite with insufficient particles will continue to elongate, unable to reach an overall activation of the matrix, increasing the energy absorbed in the tensile tests as long as the chain structure permits it. Such samples endure longer the state described in Scheme 2b, hardly enhancing the energy level of the structure up to activation and still preserving a vitreous phase. Consequently, samples with lower particles quantity that embrittled due to aging, e.g., PLA_1, PLA_0.5, and PLA_5 show a broadened post-craze-yielding behavior in Figure 3b and an enhanced energy absorption as seen in Figure 5. Unable to achieve the overall activation, the primary structure fails within the vitreous matrix phases, ending in the observed brittle failure.

## 4 Conclusions

In this work, the toughening effect of BaSO_4_ particles in PLLA is addressed, considering the hybrid segmental structure of glassy polymers and the role that physical aging plays in the mechanical performance of the composite. In these terms, we conclude that the debonding of particles locally enhances the energetic state of the primary structure, resolving the imposed strain without involving the failure of the chain network. Nonetheless, further aging lowers the energy landscape of the primary structure, hindering the effect of debonding and making the composites progressively exhibit brittle breakups. Ductile behavior endured further in time when a higher number of particles was added to the matrix due to the reduced gap between activated fractions of the structure.

Even if initially the PLLA composites showed ductile behavior, it has been evinced that the amount of particles in the matrix is critical to preserve the mechanical properties of PLLA in time. Consequently, we want to remark on the significance of testing PLLA-based composites considering physical aging in order to characterize their behavior accurately also in time, particularly if parts are expected to be stored before use. These results only depict basic mechanical performance, and tensile mechanical testing should be complemented with additional techniques mimicking the human body environment, including compression, three or four point bending, torsion, impact tests, and dynamic fatigue testing whenever a material is studied toward clinical implantation.

## Supporting Information Available

Particle morphology and distribution; DSC data collection methods; WAXS diffraction curves; particle debonding measurements; tensile test curves; SEM and SEM-BSE; and Δ*C*_p_ versus *X*_c_ (PDF)
