# Supplementary material for: Role of Inorganic Fillers on the Physical Aging and Toughness Loss of PLLA/BaSO4 Composites
Source: ACS Appl Polym Mater. 2023 Nov 1;5(11):9620–31. doi: 10.1021/acsapm.3c02112 (PMC10653123; doi:10.1021/acsapm.3c02112)
Supplement: Supplementary file 1 — ap3c02112_si_001.pdf [file ap3c02112_si_001.pdf]

Supporting Information

The role of inorganic fillers on the physical aging and toughness loss of PLLA/BaSO4 composites

Xabier Larrañaga[a], Jose R. Sarasua[a], Ester Zuza[a*]

[a]University of the Basque Country (UPV/EHU) Department of Mining-Metallurgy Engineering and Materials Science & POLYMAT Faculty of Engineering, Alameda de Urquijo s/n, 48013 Bilbao, Spain

Corresponding Author

*Ester Zuza Hernandez

Department of Mining-Metallurgy Engineering and Materials Science & POLYMAT

Faculty of Engineering, Alameda de Urquijo s/n, 48013 Bilbao, Spain

Email: ester.zuza@ehu.eus

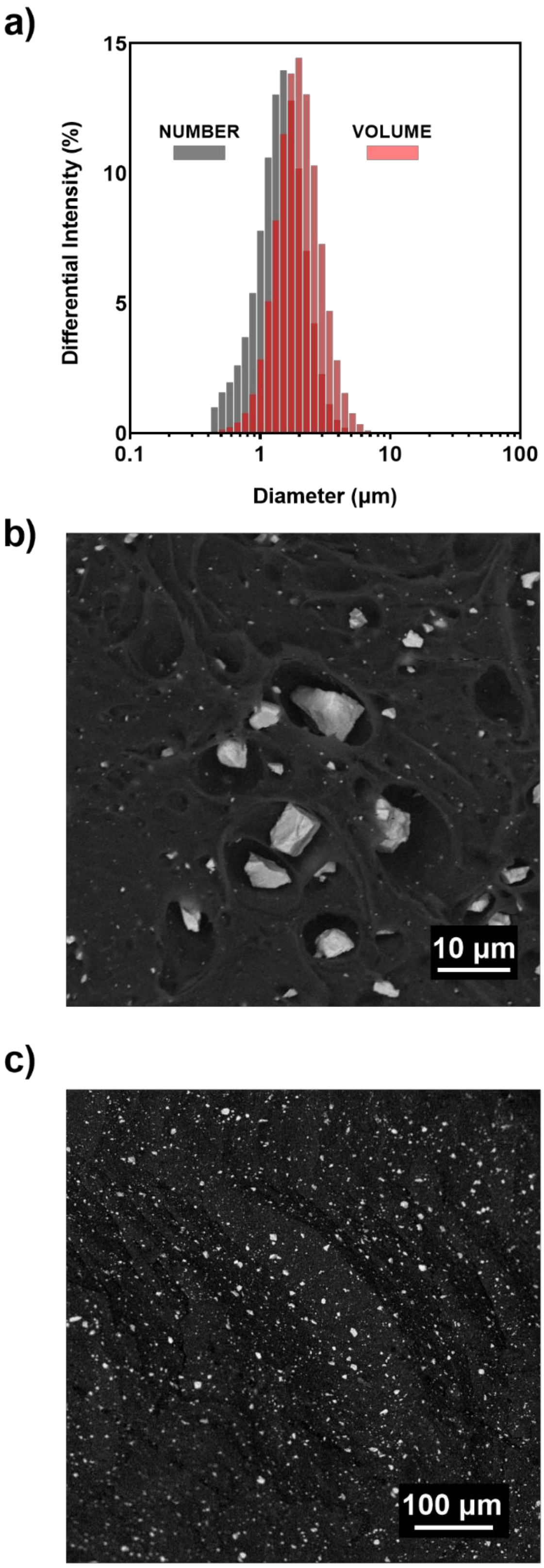

Figure S1. a) Particle size distribution in volume and number of $BaSO_4$. b) Close-up SEM -BSE image of $BaSO_4$ particles within the PLLA matrix. c) Dispersion of $BaSO_4$ particles within the PLLA matrix, SEM-BSE image.

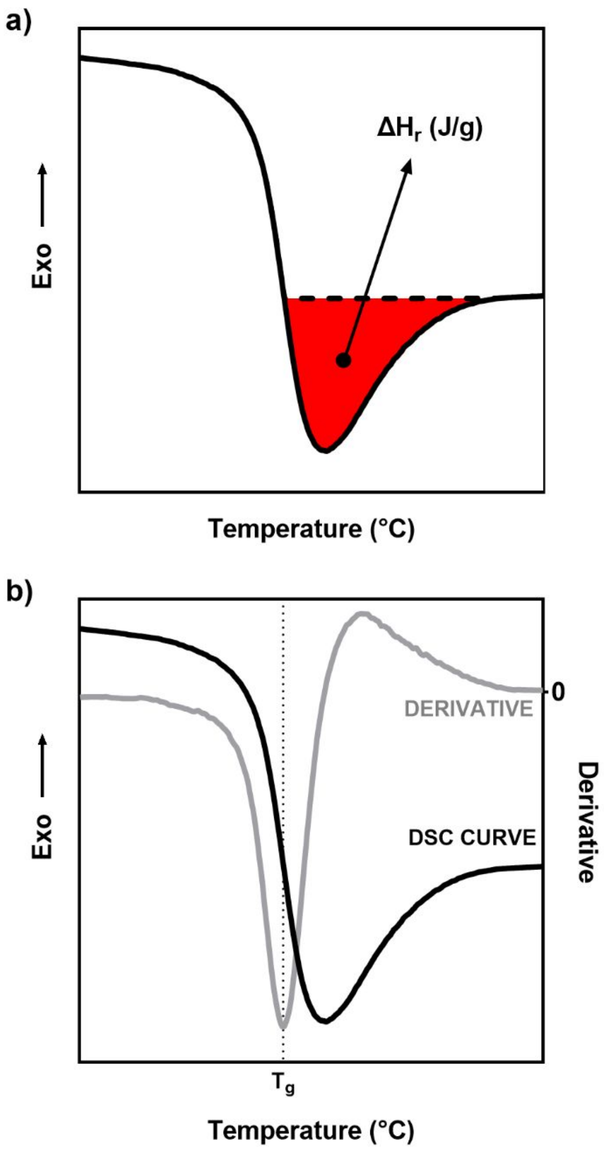

Figure S2. a) Method of obtaining the Relaxation enthalpy (ΔH_r) from DSC curves. b) Method of obtaining the glass transition temperature (T_g) from DSC curves.

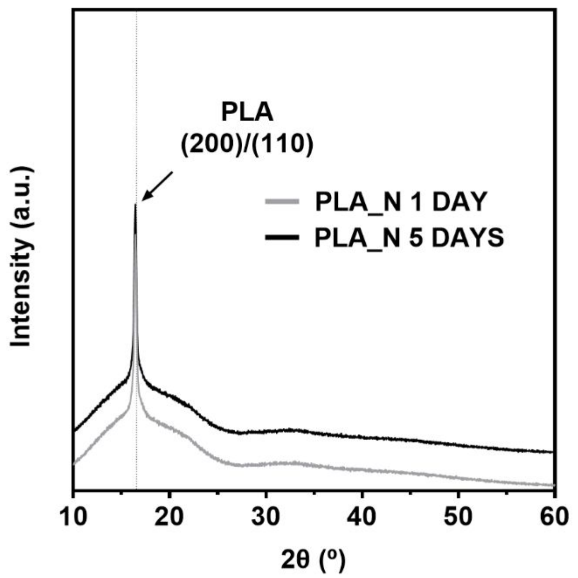

Figure S3. X-ray diffraction curve of PLA_N aged for 1 and 5 days. Differences in peak intensity come from variations in exposed area, exposure time and inclination of samples due to sharp crystal orientation.

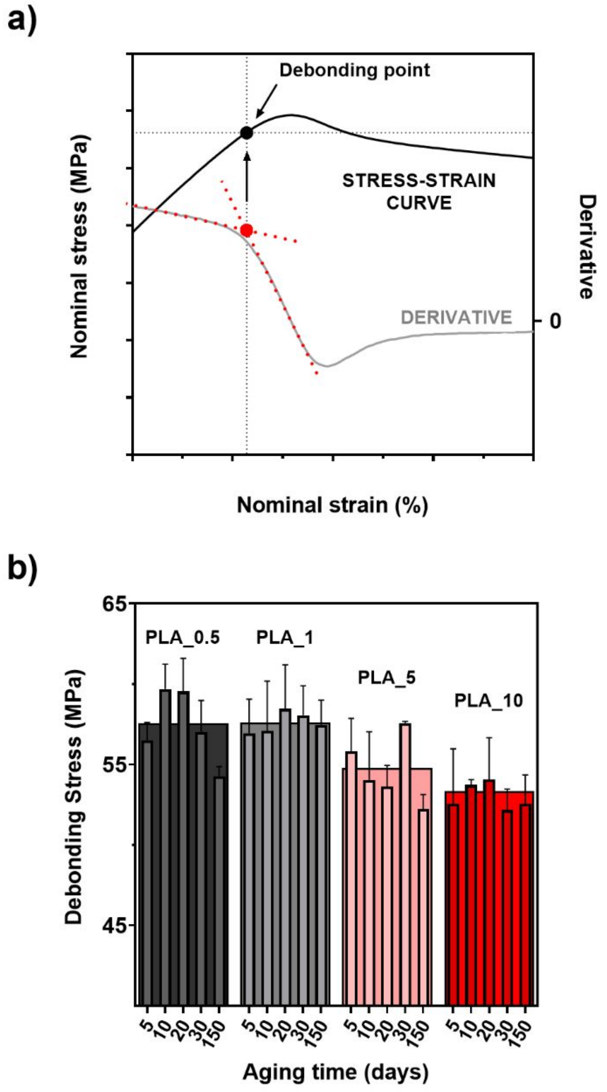

Figure S4. a) Method of obtaining the debonding point in the tensile test curves. b) Debonding stress values of all samples at every aging stage.

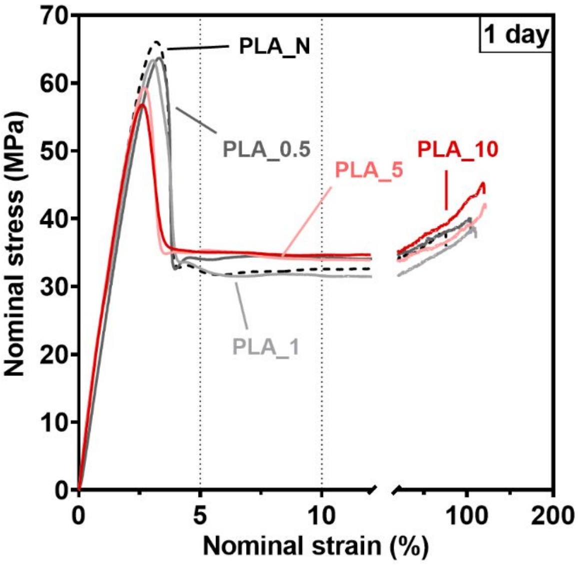

Figure S5. Stress-strain curves of uniaxial tensile tests of composite samples and PLA_N aged for 1 day as reference.

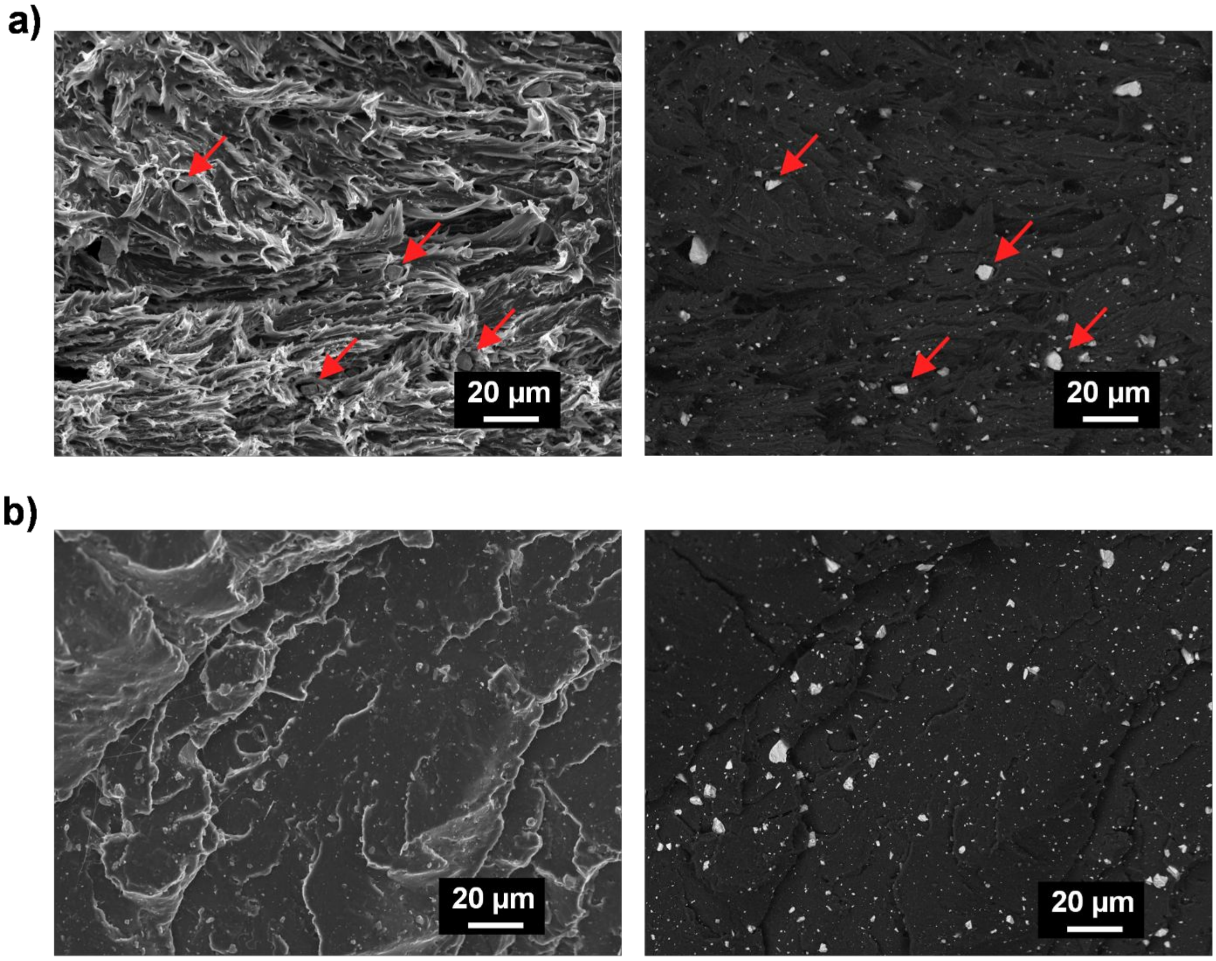

Figure S6. a) Side by side SEM (left-same of Figure 3c) and SEM-BSE (right) images of the same break surface area of ductile PLA_10, red arrows pointing towards the same particles in Figure 3c. b) Fracture surface of brittle PLA_5, SEM image in the left and SEM-BSE image in the right.

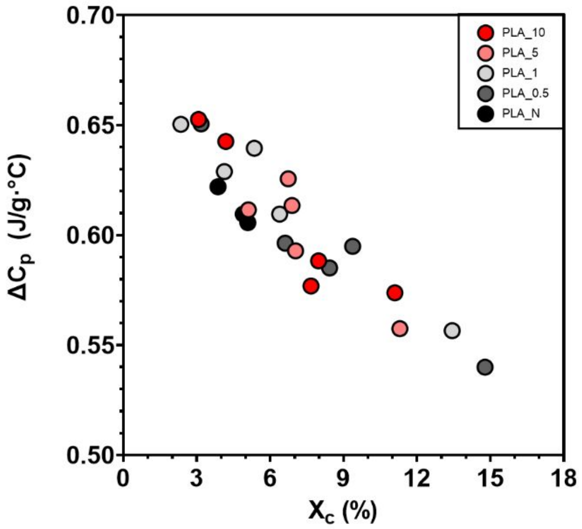

Figure S7. Change of the specific heat capacity (ΔC_p) versus the crystallinity of the sample. Symbols are colored indicating the correspondent composite system as described in the legend.
